# The Application of Predictive Modelling for Determining Bio-Environmental Factors Affecting the Distribution of Blackflies (Diptera: Simuliidae) in the Gilgel Gibe Watershed in Southwest Ethiopia

**DOI:** 10.1371/journal.pone.0112221

**Published:** 2014-11-05

**Authors:** Argaw Ambelu, Seblework Mekonen, Magaly Koch, Taffere Addis, Pieter Boets, Gert Everaert, Peter Goethals

**Affiliations:** 1 Department of Environmental Health Sciences and Technology, Jimma University, P.O. Box 378, Jimma, Ethiopia; 2 Center for Remote Sensing, Boston University, 725 Commonwealth Avenue, Boston, Massachusetts, United States of America; 3 Ethiopian Institute of Water Resources, Addis Ababa University, Akaki Kifleketema, Addis Ababa, Ethiopia; 4 Laboratory of Environmental Toxicology and Aquatic Ecology, Ghent University, J. Plateaustraat 22, 9000 Ghent, Belgium; University of Idaho, United States of America

## Abstract

Blackflies are important macroinvertebrate groups from a public health as well as ecological point of view. Determining the biological and environmental factors favouring or inhibiting the existence of blackflies could facilitate biomonitoring of rivers as well as control of disease vectors. The combined use of different predictive modelling techniques is known to improve identification of presence/absence and abundance of taxa in a given habitat. This approach enables better identification of the suitable habitat conditions or environmental constraints of a given taxon. Simuliidae larvae are important biological indicators as they are abundant in tropical aquatic ecosystems. Some of the blackfly groups are also important disease vectors in poor tropical countries. Our investigations aim to establish a combination of models able to identify the environmental factors and macroinvertebrate organisms that are favourable or inhibiting blackfly larvae existence in aquatic ecosystems. The models developed using macroinvertebrate predictors showed better performance than those based on environmental predictors. The identified environmental and macroinvertebrate parameters can be used to determine the distribution of blackflies, which in turn can help control river blindness in endemic tropical places. Through a combination of modelling techniques, a reliable method has been developed that explains environmental and biological relationships with the target organism, and, thus, can serve as a decision support tool for ecological management strategies.

## Introduction

It is important to investigate the ecological factors affecting the distribution of blackflies in order to understand blackfly ecology and their environmental dynamcis [1], [2]. Blackflies are one of the most frequently occurring aquatic taxa in tropical countries such as Ethiopia [3], [4]. These organisms are important pollution indicators of running water habitats [5], [6]. Because of their sensitivity to different environmental changes, they have been used to assess the impact of climate change and other anthropogenic activities [7]. Some species of blackflies (e.g. *Simulium damnosum*) are also known vectors of river blindness (onchocerciasis) in sub-Saharan Africa [8].

Predictive models are often applied to assess, monitor and control environmental factors of a given taxon [9], [10]. Predictive modelling is one of the most essential steps in the development of a standard habitat assessment protocol that links organisms and habitat information to environmental data [11]–[13]. Effective habitat models need to be simple, robust and at the same time biologically meaningful [14]. The goal of applying different predictive models is to simplify complex systems and to enable reliable predictions [15].

Generalized additive models (GAMs) [16] and classification trees (CTs) [17] are widely used predictive models because they are fairly simple and transparent to understand, which allow easy application into an environmental decision support system [10], [18], [19]. Such models can be useful for policy and decision-makers to improve the effectiveness of monitoring and assessment activities in different ecosystems [20].

Although linear models are attractive because of their simplicity, they often fail in addressing natural relationships between a species and biotic and abiotic variables because of their nonlinear nature [21]. Non-linear and non-monotonic relationships between the outcome and the set of explanatory variables can be meaningfully modelled using GAMs. The model accommodates non-normal data by clearly constructing the distribution as a member of the exponential family and map the relationship between the predictor and the mean of the data [22]. The main advantage of GAMs is their ability to deal with non-linear and non-monotonic relationships between the predictor and response variables because of the capability to model non-linear data using non-parametric smoothers [23]–[25].

CTs are used as an effective habitat suitability modelling technique to determine the presence/absence and abundance of species [9], [10], [18]. Genetic algorithms (GA) are one of known techniques to boost model performance and improve the accuracy and predictive power by minimizing number of irrelevant attributes [10], [26]. GA is widely used optimisation method for predictive models in the field of aquatic ecology [9], [10], [27]. Reliable CT models having best performance can be constructed when it is combined with GA [10].

The use of CT combined with GA and the application of GAMs can help to identify the major variables predicting the occurrence of Simuliidae larvae by minimizing model uncertainty [28]. In addition to the model combination, the use of environmental as well as biological predictors in the model construction is known to minimize prediction errors and ensure reliable model output [29]. Our main aim is to identify biological determinants in terms of other macroinvertebrate groups and environmental parameters, which are crucial for the presence/absence and abundance of blackflies, using GAMs and CTs combined with GAs in order to fill current knowledge gaps on the blackfly ecology, thus, leading to a better understanding of the underlying environmental factors.

## Methods

### Study area

**Figure 1:**
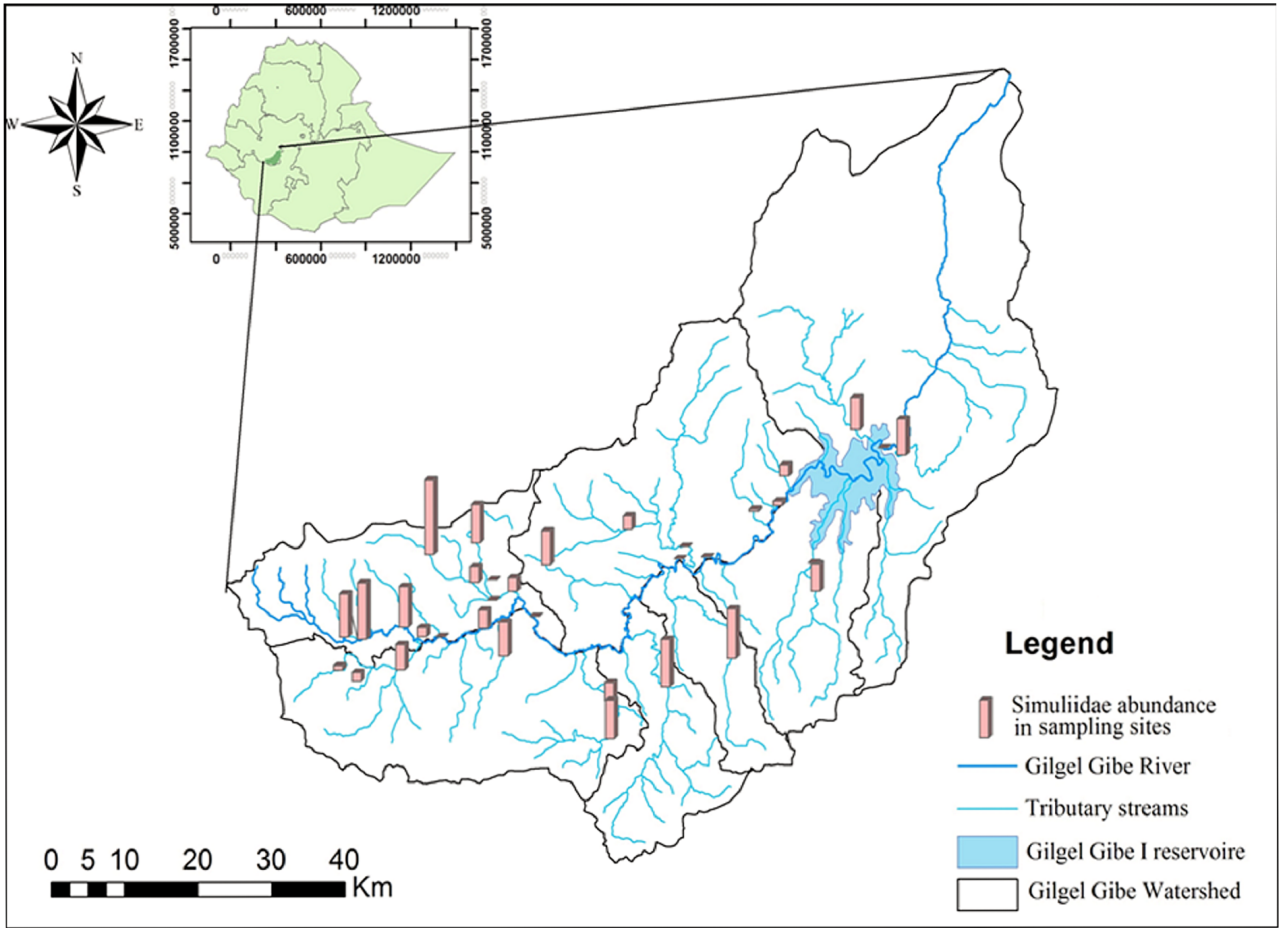
Location of the study area with bar graphs showing the abundance of Simuliidae larvae at each sampling site. The longest bar represents 33 Simuliidae individuals and the shortest one represents zero individuals.

The study was performed in the Gilgel Gibe watershed, which is part of the Omo-Gibe River basin situated in southwest Ethiopia. Simuliidae larvae are found in most of the study sites where their abundance is indicated as a bar graph in Fig. 1. The area is bounded by latitudes 7°25′ and 7° 55′ North and longitudes 36°30′ and 37° 22′ East. The watershed is mainly located in the Jimma administrative zone, which has an estimated population of over 2.5 million people (CSA, 2007). The study area receives annual rainfall in the range of 1200–2800 mm, while the altitude ranges from 1096 to 3259 m above sea level. The Gilgel Gibe watershed is located in the tropical afro-alpine ecological region.

The river basin has a catchment area of about 5371 km^2^ [10] and the sampling points are distributed along a total length of 186 km from the source to an area further downstream of the Gilgel Gibe hydropower reservoir. During the last 20 years, the Gilgel Gibe river basin has received increased attention from the Ethiopian government for implementing development projects, specifically for hydropower generation [10]. The Gilgel Gibe watershed has many rivers and streams from fast flowing forest streams to stagnant waters and even marshlands. Jimma region is known to have a high forest cover compared to other parts of the country though this is currently dramatically changing due to resettlement and agricultural expansion [30]. The sampling sites and the distribution of Simuliidae larvae are shown in Fig. 1.

### Data collection

Data was collected from different rivers in the Gilgel Gibe river basin. About 180 samples were collected from 34 study sites in five sampling campaigns. The governing authority for rivers in Ethiopia is the Ministry of Water, Irrigation and Energy. However, to undertake this study, permission from the Ministry was not required because none of the sampling sites were protected or needed special permission. Therefore, obtaining the permission from Jimma University was sufficient to collect samples from each of the sites as they are authorized to undertake such activities.

Coordinate points of each of the sampling sites are 36°39′7.853”E & 7°33′46.697”N, 36°40′12.455”E & 7°34′51.858”N, 36°40′12.455”E & 7°34′51.858”N, 36°40′52.675”E & 7°36′2.288”N, 36°43′59.173”E & 7°36′51.886”N, 36°44′42.679”E & 7°36′14.303”N, 36°43′43.812”E & 7°34′4.363”N, 36°46′28.268”E & 7°36′9.447”N, 36°45′53.931”E & 7°42′5.768”N, 36°49′19.443”E & 7°42′55.442”N, 36°49′9.523”E & 7°39′57.136”N, 36°50′3.572”E & 7°40′16.315”N, 36°50′33.787”E & 7°38′50.039”N, 36°50′44.889”E & 7°38′50.549”N, 36°49′51.072”E & 7°36′50.987”N, 36°51′20.99”E & 7°34′52.595”N, 36°54′31.28”E & 7°41′22.464”N, 36°53′40.939”E & 7°37′38.038”N, 37°0′30.67”E & 7°43′58.139”N, 36°59′12.895”E & 7°31′37.667”N, 36°59′16.273”E & 7°29′11.822”N, 37°3′18.938”E & 7°32′38.206”N, 37°4′16.022”E & 7°41′49.515”N, 37°4′42.857”E & 7°42′43.66”N, 37°6′23.256”E & 7°41′58.186”N, 37°8′16.153”E & 7°34′46.464”N, 37°9′50.927”E & 7°45′13.589”N, 37°12′8.41”E & 7°47′45.456”N, 37°11′36.415”E & 7°45′46.457”N, 37°14′17.279”E & 7°41′30.491”N, 37°14′28.6”E & 7°39′31.672”N, 37°17′24.483”E & 7°51′14.785”N, 37°19′24.012”E & 7°49′54.218”N, and 37°20′26.46”E & 7°49′19.344”N 36°50′44.889”E 7°38′50.549”N.

Each campaign was carried out at a six-month interval and samples were taken during dry and wet seasons. The study sites were selected *a priori* based on the criteria of accessibility, geographical distribution, and existing variations of natural and anthropogenic activities. The collected data are categorized into three parts: a) physical-chemical data, b) macroinvertebrate data, and c) physical habitat (physiographic) data (e.g. water depth, water width, river bed, vegetation cover, etc).

#### Physical-chemical parameters

Temperature (°C), conductivity (µS.cm^−1^), pH (-), oxygen saturation (%) and turbidity (NTU) were measured onsite at each sampling location using hand electrodes. Five day biochemical oxygen demand (BOD_5_) (mg.L^−1^), nitrate-nitrogen (described as nitrate) (mg.L^−1^), ammonium-nitrogen (described as ammonium) (mg.L^−1^) and orthophosphate-phosphorus (described as phosphate) (mg.L^−1^) were analysed in the laboratory according to standard methods [31].

#### Physiographic and habitat data

The water body width, water depth and flow velocity were assessed according to Ambelu (2009). The riparian vegetation, river sinuosity, river bank status and embeddedness were estimated using US-EPA habitat assessment protocol [32].

#### Biological data

Larvae of Simuliidae and other macroinvertebrates were collected using the kick-sampling technique which consists of a D-frame net having a mesh size of 300 µm diameter (Ambelu et al., 2010). Kick sampling was performed along a 10 meter stretch of the river for five minutes including all the microhabitats within the sampling reach [33]. During sampling, the river bed was thoroughly disturbed by kicking with the feet in order to dislodge the macroinvertebrates from the substrate. All substrates in the sampling reach were thoroughly checked to capture organisms attached to it. Within the five minutes of kick sampling, all the possible areas of pool, riffle, edge and center were sampled. After sampling, macroinvertebrates were sorted alive onsite and preserved in 70% ethanol. In the laboratory, the sorted macroinvertebrates were identified to family level using a stereo-microscope and the identification keys [34], [35].

### Modelling procedures

The modelling was performed using two groups of predictors, namely environmental and macroinvertebrate data. The summary statistics of the response variables in relation to Simuliidae larvae are presented in Table 1 and Table 2. All the environmental variables used were log transformed (except pH) and a square root transformation was done for all macroinvertebrate data. For the application of GAM, a transformation was necessary in order to achieve a uniform distribution [24].

**Table 1:** The minimum (Min), 1^st^ quartile (1st Qu), median, mean, 3^rd^ quartile (3rd Qu), maximum (Max) and standard deviation (StDv) of environmental predictors used to analyse Simuliidae occurrence.

Environmental variables	Min	1^st^ Qu	Median	Mean	3^rd^ Qu	Max	StDv
Altitude (m)	1625	1698	1742	1772	1788	2488	121.43
Vegetation (score out of 20)	2	6	10	10.3	13	20	5.3
Water temperature (°C)	13.9	18	19.68	19.8	21.1	27.5	2.42
Width (m)	0.6	3	6	8.7	10	43	9.24
Depth (m)	0.01	0.25	0.43	0.6	0.7	2.5	0.43
Velocity (m/s)	0.005	0.22	0.44	0.5	0.7	1.8	0.32
Flow rate (m3/s)	0.001	0.26	1.05	2.8	3.15	27.36	3.84
Sinuosity (score out of 20)	6	10	14	14	18	20	4.13
Distance from source (m)	2	12.5	19	29.8	29	154	34.9
Embeddedness (score out of 20)	3	10	16	14.4	18	20	4.91
River bank status (score out of 20)	4	12	15	14.2	18	20	4.4
pH	5.3	7.02	7.4	7.4	7.7	8.5	0.47
Conductivity (µS/cm)	27.1	80	100	114	130	455	57.9
DO (mg/L)	0.34	5.83	6.7	6.4	7.31	9.3	1.54
BOD (mg/L)	0.21	1.6	2.5	4.1	3.6	80	6.34
Phosphate (mg/L)	0	0.03	0.16	0.4	0.5	4.47	0.57
Nitrate (mg/L)	0.01	0.402	1.2	1.4	1.9	6.156	1.13
Ammonium (mg/L)	0.002	0.05	0.22	0.5	0.8	3.13	0.62
Simuliidae (count)	0	0	0	5.297	4	150	16.08

**Table 2:** The median, mean, 3^rd^ quartile (3rd Qu), maximum (Max) and standard deviation (StDv) of macroinvertebrate (MI) variables used to predict Simuliidae abundance and presence-absence.

MI variables	Median	Mean	3^rd^ Qu	Max	StDv
Aeshnidae	0	1	0	10	1
Anthomyidae	0	9	0	74	19
Baetidae	5	15	19	150	25
Belostomatidae	0	1	0	27	3
Caenidae	4	11	14	155	21
Chironomidae	6	11	12	125	17
Coenagrionidae	4	11	13	88	17
Corduliidae	0	1	0	20	2
Corixidae	0	2	2	50	6
Dytiscidae	0	4	2	150	17
Elmidae	0	1	1	43	3
Ephemerellidae	0	1	0	53	3
Glossiphonidae	0	1	0	47	3
Glossosomatidae	0	1	0	62	5
Gomphidae	0	1	2	22	3
Gyrinidae	0	1	0	23	2
Heptagenidae	0	3	2	110	10
Hydrophilidae	0	1	1	26	2
Hydropsychidae	3	15	19	150	26
Libellulidae	1	5	4	100	11
Naucoridae	0	1	1	31	3
Nepidae	0	0	0	4	1
Notonectidae	0	1	0	82	5
Protoneuridae	0	3	3	37	6
Sphaeriidae	0	1	1	41	4
Tipulidae	0	0	0	8	1
Unionidae	0	1	0	21	3

The minimum and the 1^st^ quartile values are not presented in the table because all were zero.

#### Generalized additive models

GAMs were applied in order to define the set of the environmental parameters and macroinvertebrate taxa that best described the habitat condition of Simuliidae and presence-absence. Additive models are a nonparametric alternative for the more conventionally used generalized linear models (GLMs). GLMs have been frequently used in ecology (Guisan et al., 2006) and are defined by
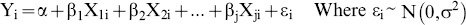
(1)


The Y*_i_* is the response variable, and X*_i_* represent the explanatory variable(s). The residuals (ε*_i_*) capture the unexplained variation in the data, which is assumed to be normally distributed with a mean value of 0 and variance σ^2^. The parameters α and β represent the intercept and slope of the regression respectively. If multiple explanatory variables are used, the number of products between β and X*_i_* is equal to the number of explanatory variables. (1) can be further conceptualized as
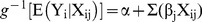
(2)


Where g^−1^(·) is a local scoring algorithm that specifies the link function between the expected value of Y*_i_* and the explanatory variables. A GAM is defined by
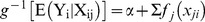
(3)


The Y*_i_* is the response variable, X*_i_*β represents the intercept of the regression equation, *f_j_*(*x_ji_*) is a smooth function of the *j* ^th^ explanatory variable, *i* = 1, …, n, is the number of observations.

The number of knots affects the amount of smoothing applied to the data [36]. A smoother with two knots is linear, has little variability and may be biased since there is only one piecewise function [37]. Increasing the number of knots allows more flexibility, but may result in over-fitting. For smaller data sets, below 30, three knots is a good starting point. [37] report that a number of four to five knots is appropriate for most applications. In our analysis, the number of knots for the smoothing curves was fixed to five for macroinvertebrate analysis and 10 for environmental variables as the number of records per substance in our training dataset varied from below 30 to more than 100.

The ‘mgcv’ library in the R statistical software [38] was used to select the GAMs smoothing predictors following the method proposed by Wood and Augustin [36]. The individual models cannot be tested for significance using the P-values provided by ‘mgcv’ library since the true number of degrees of freedom is unknown (Giannoulaki et al., 2008; Wood, 2012). Each model fit was analyzed by the level of deviance explained (0–100%; the higher the better), and the unbiased risk estimator (UBRE) in which the lowest value is considered as the best model performance indicator. The degree of smoothing was also chosen based on the observed data and the generalized cross validation method suggested by [25] and incorporated in the ‘mgcv’ library. To avoid the over-fitting problem, the effective degree of freedom of each model count in the GCV score was increased by a factor of γ = 1.4 [39].

To increase the model performance and decrease the collinearity problem, independent variables were eliminated [22], [23], [25] and the best model was chosen based on a stepwise backward selection method. Specifically, models were compared using the estimated UBRE and percent deviance explained, the environmental variables were ranked and selection of the final model was based on the minimization of the above criteria. Following the recommendation forwarded by Wood (2001), during model fitting manual elimination of attributes was done when all of the following three criteria are met: the estimated degree of freedom of the model term is closer to 1; the plotted confidence band from the model term include zero everywhere; and URBE score is dropped when the model term (attribute) is eliminated.

The relationship between Simuliidae larvae and the predicting variables (e.g. pH) with the *i^th^* observation in the data, smooth function *s*(), constant *a*, and residual error *i* is represented by:
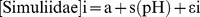
(4)


Therefore a model with *n* smooth functions (predictor variables) in this relationship can be generalized to:
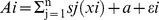
(5)


The *i^th^* Simuliidae abundance in the data set is *Ai*. *sj*(*xi*) is the smooth for the *j^th^* variable and gives the value of this smooth for the *i^th^* observation. *i* is the residual error for this observation and *a* is a constant.

#### Classification tree combined with genetic algorithms

First, the model was developed based on CT using all input predictors, while in a next steps the CT was combined with a genetic algorithm, which was used to select the most relevant input variables. CTs [17], [40] predict the value of a discrete dependent variable with a finite set of values (called classes) from the values of a set of independent variables (called attributes), which may be either continuous or discrete. The J48 algorithm with binary splits was applied to induce CT. There are a variety of algorithms to build classification trees that share the desirable quality of interpretability. A well-known and frequently used algorithm is the C4.5 which is a java reimplementation of the J48 algorithm in the WEKA machine learning package [41]. The dependent variable (output value) consisted of the presence-absence of Simuliidae larvae whereas the independent parameters were the physical-chemical and MI larvae predictors (Table 1 and 2).

Different folds of cross-validation were tested for the training and validation of CTs. The maximum stability and model performance of CT was maximized using a 10-fold cross-validation in terms of percentage of correctly classified instances (%CCI) and Kappa statistics (*k*). In the 10-fold cross-validation, the original data were randomly partitioned into 10 subsamples of approximately equal size using WEKA default settings. In addition, the default values of the J48 algorithm with binary split were used to find the most important explanatory variables for the prediction of Simuliidae.

The next step was the application of the GA search method on the CT to select the best explanatory variables for the Simuliidae larvae. GAs follow the principle of “*survival of the fittest*” which begin with a population of randomly generated chromosomes that advances towards the selection of better chromosomes [42]–[44]. Following the principle of natural selection, the population undergoes evolution with successive generations. During this process, chromosomes in the population are rated for their fitness and consequently a new population of chromosomes are formed depending on the applied selection method.

During CT model development, wrapper subset evaluator was used on J48 learning algorithm in which the attributes (variables) are evaluated by using accuracy estimations [45]. During GA application, we used 20 chromosomes as initial population that evolved through a maximum of 20 generations [26]. Default settings of Weka machine learning algorithm was used for crossover and mutation probability which is 60% and 3.3%, respectively. Before the GA application, the dataset was randomized and then attributes were selected. After the selection of the successful chromosomes, CTs were run seven to ten times to each subset (chromosome) after randomization to check the stability of the model. The subsets of selected attributes by GAs (chromosomes) that showed the lowest standard deviation, based on %CCI and *K*, were retained. In addition, attributes that appeared most frequently in the subsequent GA application were finally used for CT-GA model development.

## Results

### GAM output

**Figure 2:**
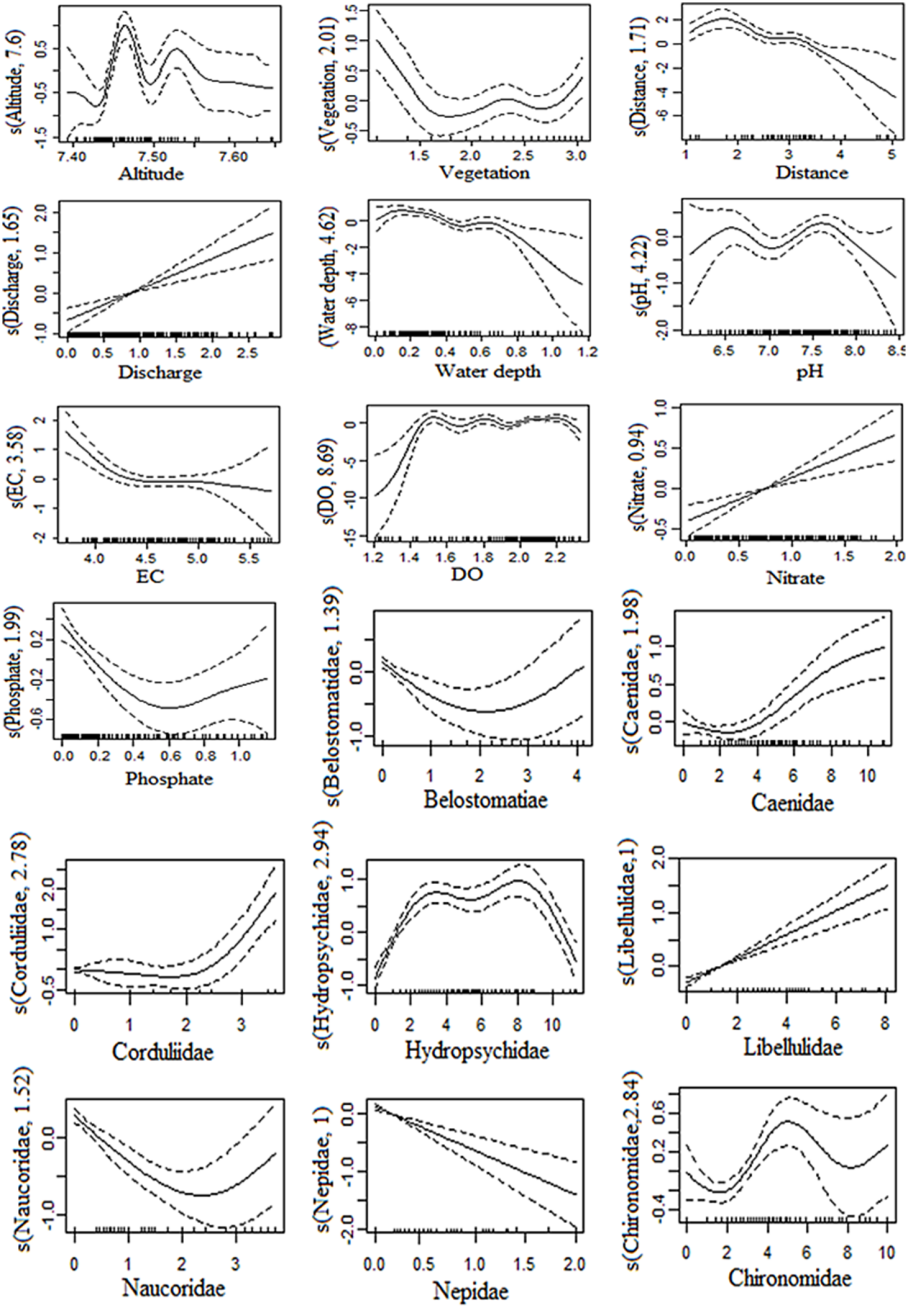
Smooth plot of the GAM output of the selected environmental and macroinvertebrate predictors showing their relationship with Simuliidae larvae and the fitted nonparametric terms with 95% confidence interval (dashed lines). The y-axis is scaled to zero and the rug plot on the x-axis indicates number of observations.

Using the abundance of the response variable (Simuliidae), 11 environmental predictor variables were obtained from the model after a backward stepwise elimination of the terms. The selected variables significantly contributed to the prediction of the Simuliidae larvae (Table 2). All eliminated variables had a very low value of estimated degrees of freedom and had non-significant p-values. The GAM has an adjusted R^2^ of 0.62 and the total deviance explained was 62% and the un-biased risk estimator (UBRE) score was 0.345. The relationship between environmental attributes selected by GAMs and the Simuliidae larvae is shown in Fig. 2.

However, when GAM prediction of the Simuliidae larvea with its presence-absence data is made, only three environmental predictors (distance, flow velocity and water depth) were selected with significant prediciton (p-value<0.01). The estimated degrees of freedom for the three environmental predictors were 2.43, 2.06 and 1.51, respectively. The EBRE score, adjusted R^2^, and percent deviance explained were respectively −0.462, 0.323 and 33.9.

Among the 27 macroinvertebrate predictors, eight were selected by the GAMs. After backward stepwise selection of the predicting variables of macroinvertebrate families, those which showed significant predicting power were fitted as shown in Fig. 2. The presence-absence of the Simuliidae larvae was also predicted with GAMs and only four macroinvertebrate predictors (Beatidae, Dytiscidae, Hydropsychidae and Libeluliidae) were selected as important variables. All four variables showed a significant (p-value<0.05) contribution to the model and have an R^2^ adjusted = 0.58, percent deviance explained = 63, and UBRE score = 0.243.

### CT-GA output

**Figure 3:**
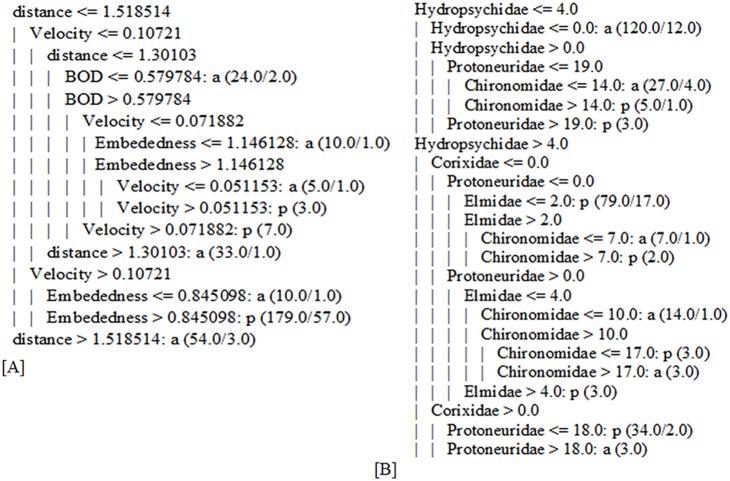
Classification tree constructed by the most frequently selected environmental [A] and macroinvertebrate [B] predictors using genetic algorithm predicting the presence (p) and absence (a) of Simuliidae larvae.

Classification tree models were built using a genetic search algorithm. Prior to the selection of the environmental attributes, the classification tree was built. The tree size was 67 with 34 leaves whereas the %CCI and *k* were 69.4±1.3 and 0.38±0.03, respectively. During the application of the genetic search algorithm, the distance of the sampling site from the source of the river appears in all the successful chromosomes. Whereas the flow velocity and embeddedness appears nine times, river bank status and DO appear seven times, BOD and ammonium appear four times, electrical conductivity (EC), flow rate and water depth appear three times, pH and nitrate appear only one time from the ten independently identified subset of attributes (chromosomes). Finally, using the most frequently selected attributes (four to ten times), a classification tree model was built. The model indicated that the presence or absence of Simuliidae is primarily determined by the distance of the site from the stream source. According to the model, the Simuliidae community are often absent for sites which are 32 km far from the source. In addition, Simuliidae is absent for sites whose flow velocity is 0.125 m/s (Fig. 3).

**Figure 4:**
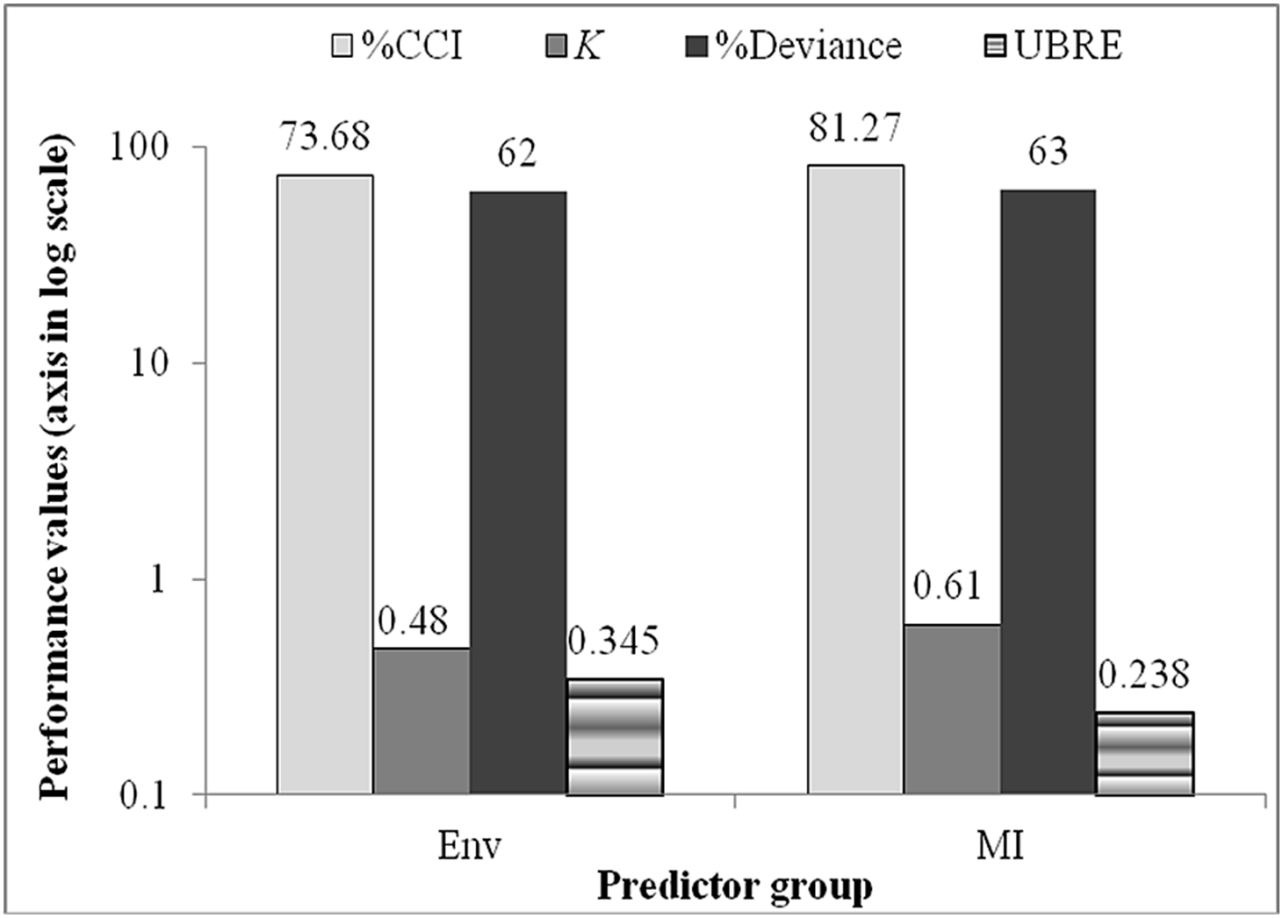
Model performances of GAMs and classification trees based on environmental (Env) and macroinvertebrate (MI) predictors. %CCI = percent correctly classified instances, K = kappa statistics, UBRE = unbiased risk estimator.

Before the application of GA on the CT, all 28 macroinvertebrate variables were used and the average performance in terms of %CCI and *K* was 78.26±0.02 and 0.53±0.02, respectively. After the application of GA, each chromosome or group of successful macroinvertebrate variables picked by the GA showed an average %CCI and *K* of 80.2–82.46 and 0.60–0.65, respectively (Fig. 4).

In each chromosome five to nine macroinvertebrates were chosen by the GA to predict the presence of Simuliidae. Corixidae (9 times), Hydropsychidae (9 times), Protoneuridae (8 times), Chironomidae (8 times), and Elmidae (6 times) were the most frequently selected macroinvertebrate variables. Glossosomatidae, Aeshnidae, Gyrinidae, Libellulidae, Nepidae, Belostomatidae, Caenidae, Dytiscidae, Hydrophilidae, Spheariidae, Tipulidae, Ephemerellidae, and Anthomyidae appeared rarely (one to two times) among the ten selected chromosomes. The other macroinvertebrates were not selected by GA. The CT model, constructed with the most frequently selected macroinvertebrate predictors by GA, is shown in Fig. 3. The model indicated that among the macroinvertebrate communities, Hydropsychidae, Corixidae, Protoneuridae, Chironomidae and Elmidae were the major determinants of the presence and absence of Simuliidae larvae.

## Discussion

Bio-environmental factors that are influencing blackflies distribution in the Gilgel Gibe watershed has been identified using combined modelling techniques. This approach enabled us a better identification of the suitable habitat conditions or environmental constraints for Simuliidae larvae. Characterizing and modelling the distribution and abundance of taxa is one of the major tasks of ecologists [46]. The availability of reliable environmental dataset obtained from wider area of sampling sites for an extended period of time often encourages prediction of taxa to identify the environmental requirements so that their distribution can be inferred. This is especially helpful for the prediction of species distribution over large unsampled areas and for reducing sampling costs. In addition, the model output could provide important information for decision support of environmental management systems. Here, we have used two well-established habitat suitability modelling techniques in order to identify important predictors that can explain the abundance and occurrence of Simuliidae larvae.

Simultaneous modelling of Simuliidae using GAMs and CTs has enabled the identification of the most important environmental and macroinvertebrate predictors. Among the environmental predictors, distance from the source, river discharge, water depth, river bank status, electrical conductivity and nitrate concentration were selected by both modelling techniques as important variables determining the occurrence and abundance of black flies in the region.

The GAM outputs indicate that the model performance indicators between the presence-absence of Simuliidae larvae significantly differ from the abundance prediction. The number of selected predicting variables (both environmental and macroinvertebrate predictors) was fewer for presence-absence compared with the Simuliidae abundance. Except for flow velocity, the other environmental presence-absence predictors were also identified by GAM during abundance prediction. A previous study done by Barry and Welsh (2002) also has indicated that the model pattern of presence-absence of a species, conditional on the covariates, is markedly different from the pattern of abundance.

We therefore determined that the abundance of Simuliidae increases with increasing river flow rate, nitrate concentration and flow velocity. Nevertheless, Simuliidae abundance regularly decreases with increasing distance of the sampling site from the source, electrical conductivity of the water, water depth and phosphate concentration. The other environmental predictors like altitude, vegetation cover, river bank status and DO concentrations show irregular patterns with regard to the abundance of Simuliidae. The optimum pH condition for Simuliidae larvae abundance was found to be approximately between 6.5 and 7.7. Regarding the selected macroinvertebrate predictors, the occurrence of Libellulidae, Baetidae, and Caenidae promotes the availability of Simuliidae larvae in the river system. However, higher abundances of Hydropsychidae, Belostomatidae, Naucoridae and Nepidae could reduce the availability of the dependent variable, i.e. Simuliidae larvae. It has been found that the GAMs prediction using macroinvertebrate communities showed better performance (in terms of UBRE, adjusted R^2^ and percent deviance explained) than the environmental predictors.

Clear model results were obtained when classification tree models were supported by a genetic search algorithm to select environmental and macroinvertebrate predictors of Simullidae larvae. The application of GA to CT significantly improved the model performance as well as the clarity of the decision tree. The decision tree model without the application of GA was complicated to understand and describe due to its large tree size. Recently [10], [19], [26] have also improved clarity of their classification tree models by applying GAs. However, those authors and many others [4], [26], [47]–[50] are often using model boosting mechanisms such as bagging and boosting, together with the use of attribute selection tools (GA, greedy stepwise algorism) rather than combining the model with robust statistical techniques like that of GAMs. Based on the given data set, the CT-GA has given clear environmental predictor values for which the Simuliidae larvae could be present or absent. The majority of environmental and macroinvertebrate predictors selected by GAM were also identified by GA as important predictors of the presence-absence of Simuliidae larvae. The two modelling techniques (GAMs and CT-GAs) showed reliable predictors which can be very useful for understanding the distribution of Simuliidae larvae and, thus, controlling the vector of onchocerchiasis. On the other hand, both the GAMs and CT-GAs models have indicated that Simuliidae larvae may be an important water quality indicator in head waters (with shorter distances from the source), shallow and fast flowing rivers.

Vector control and patient treatment is a major component of the Onchocerciasis control program and is based on routine aerial application of larvicides. This is found to be very expensive to implement in many developing countries like Ethiopia and Ghana where the disease is endemic [51]. Therefore, our model outputs could indicate an alternative means to control the disease vector larvae based on environmental management and biological control mechanisms. Environmental management and biological control of the disease vector may be a much more effective strategy than the use of pesticides to overcome the residual effects of chemical applications to the different environmental compartments. The GAMs and CT-GA have been successfully applied to identify the environmental variables and macroinvertebrates that can play a detrimental role in the elimination of Simuliidae larvae from the river system. GAMs and classification trees can even indicated which areas should be focused on for insecticide application if it becomes a choice of vector control. Based on the selected variables it should be possible to map the sites where Simuliidae is present. Such mapping has been proposed by [51] to help control the occurrence of onchocerchiasis.

According to GAMs, one of the major environmental management strategies that could be applied is minimizing the flow velocity and increasing the water depth so that the abundance of Simuliidae larvae would be minimized or eliminated. This could be achieved by slowing down the flow in the highlands which would reduce the flow velocity and increase the water depth. This procedure could benefit communities affected by Onchocerchiasis because they could utilize the additional water for irrigation to ensure food security. This is a very relevant issue in arid tropical countries where farmers cannot dependant on rain water only but need river water for irrigation. The model outputs based on macroinvertebrate variables could be an important indication for when biological control methods need to be applied to Simuliidae. However, it is recommended to further study the biological relationship of the identified macroinvertebrates and Simuliidae to effectively apply such biological control of Simuliidae.

## Conclusion

In conclusion, the combination of GAMs and CT-GA techniques has led to the identification of suitable habitat conditions of Simuliidae larvae and the macroinvertebrate families, which are crucial for their existence or disappearance. Such models are important for conservation purposes as well as for disease vector control in the tropics because they can be used to eliminate the suitable environmental conditions of the target organism [52]. Accurate representation of species distribution models derived from sampled data is essential for tropical ecosystem management purposes. Effective prediction of the habitat suitability of Simuliidae larvae has been obtained by the combined application of GAMs and CT-GAs. Through this modelling approach, a more reliable ecological assessment and Onchocerchiasis disease vector control could be achieved based on environmental management and biological control techniques. The results may lead to improved vector control methods using habitat modification techniques and site specific application of pesticides.
